# Tissue Banking: Relationship with Blood Donor and Organ Donor Card Status

**DOI:** 10.5402/2012/475729

**Published:** 2012-03-05

**Authors:** Kenneth D. McKenzie, Patricia E. Fitzpatrick, John D. Sheehan

**Affiliations:** ^1^School of Public Health, Physiotherapy & Population Science, University College Dublin, Dublin 4, Ireland; ^2^Department of Psychiatry, Mater Misericordiae University Hospital, Dublin 7, Ireland

## Abstract

Understanding the relationships among altruistic health acts may serve to aid therapeutic research advances. In this paper, we report on the links between two such behaviours—donating blood and carrying an organ donor card—and willingness to donate urological tissue to a tissue bank. Reasons for the differential willingness to do so are examined in this paper. A systematic sample of 259 new and returning attendees at a tertiary urology referral clinic in Ireland completed a self-report questionnaire in an outpatient setting. In addition to demographic details, details of known diagnosis of malignancy and family history of cancer; attitudes to tissue donation for research purposes were gauged using a 5-point Likert scale. Both blood donors and organ donor card carriers were more likely to be willing to donate tissue for research purposes. Blood donors were more likely want to know their overall results in comparison to nonblood donors and want their samples to be used for nonprofit research. Our hypothesis that being a blood donor would be a better predictor to donate urological tissue than being an organ donor card carrier borne out by the trends reported above.

## 1. Introduction

Working to increase willingness to donate tissue collected for diagnostic purposes to a tumour bank has been highlighted as an important factor in clinical research [1]. Understanding the determinants of altruistic behaviours in health research has long been featured as a research question [2]. In this paper, we look at the link between the individual altruistic acts of blood donation and organ donor card carrying, and willingness to donate urological tissue to a tumour bank.

The dominant model of understanding the motivations around blood donation has been the theory of planned behaviour (TPB) [3], which assumes that intention is the most contiguous determinant of action, and that intention is determined by perceived behavioural control, attitude, and subjective norm. Key demographic findings of a greater likelihood of donating blood include being male [4] and comparative youth: cohorts aged 56 and over tend to be underrepresented in blood donors [5]. The main psychological determinants have been found to be altruism [2], perceived control, and self-identity as a blood donor. Perceived control is a stronger predictor of motivation to donate blood ahead of positive attitude [6, 7], where there is a stronger intention to donate if the prospective donor thinks that donation clinics are accessible. Self-identity as a blood donor develops with repeated donations thus strengthening self-descriptions as a donor [4]. Knowledge of blood donation has not been found to predict intention to donate blood [8].

There has been an emphasis on situational rather than TPB variables in the study of predicting intentions to donate organs. Higher information levels on organ donation and knowing someone who carried an organ donor card were linked with the carrying of an organ donor card [9]. Relationship between a potential donor and a recipient, and the type of organs to be donated, have both been reported to predict willingness to donate [10]. Prior utilisation of the health care system has also been linked to willingness to donate [11].

We hypothesised that both blood donation history and carrying of an organ donor card would predict willingness to donate tissue to a tumour bank. Specifically, we predicted that there would be a greater effect for blood donation over carrying an organ donor card, as the habit and accrued social status of blood donation are distinct to the anonymous and unpractised behaviour of carrying an organ donor card. We also examined the extent to which the willingness to donate tissue to a tumour bank was accompanied by conditions around the purposes to which the donated tissue was put; the desire to know individual and overall results of research carried out on one's donated sample; whether the research was for profit or not.

## 2. Methods

A systematic sample of 259 male patients attending the urology clinics in two tertiary referral urological centres participated in the study. The age range of the sample was 20–87 years old, (mean 55 years old). The patients were both new and returning attendees.

The study was approved by UCD Research Ethics Committee, with informed consent obtained from all participants.

The questionnaire comprised basic demographic items, details of known diagnosis of malignancy, and family history of cancer. Attitudes to tissue donation for research purposes were gauged using a five-point Likert-type scale.

The response rate was 98%. Further details of methods have been previously published [12].

## 3. Results

Table 1 shows the responses to a series of statements. The odds ratios are adjusted for age and show how the chances of being willing to donate urological tissue change under the conditions of ever having been being a blood donor and carrying an organ donor card. Blood donors were almost three times more likely willing to donate tissue to a tumour bank than nonblood donors. Although just failing to reach significance, there is a similar magnitude of increased willingness to donate tissue vis-a-vis organ donor card carriers and those who did not carry an organ donor card.

With regard to tissue samples after donation, organ donor card carriers are two and a half times more likely than nonorgan donor card carriers to having no objection to their tissue being used for any research activity. Both blood donors and organ donor card carriers were more likely want to know their individual results, but this tendency was stronger in blood donors. Blood donors were also more likely to want to know their overall results in comparison to non-blood donors and to want their samples to be used for non-profit research.

## 4. Discussion

Both acts of blood donation and carrying an organ donor card are positively linked with greater willingness to donate urological tissue to a tumour bank. It should be noted that willingness to donate was very high, irrespective of the respondent's status on both ever having donated blood and currently carrying an organ donor card.

Our hypothesis that being a blood donor would be a better predictor to donate urological tissue than being an organ donor card carrier borne out by the trends reported above.

It is plausible that repeated acts of blood donation are likely to strengthen a sense of medical altruism in the person's identity when compared with the once-off and less public act of carrying an organ donor card. As a result, the trends for wanting the tissue to be used for non-profit research, and for permitting the tissue to be used for any purpose, are more understandable.

## Figures and Tables

**Table 1:** Responses to statements, as *n* (%), concerning the willingness to donate tissue samples for medical research and the use of tissue samples.

Question	Strongly agree	Agree	Unsure	Disagree	Strongly disagree
*Donation*					
If I was ill and had to have a biopsy sample taken from my prostate gland or had prostate tissue removed at an operation, I would be willing to donate tissue samples from my prostate gland for research purposes	115 (45.3)	97 (39.2)	34 (13.4)	7 (2.7)	1 (0.4)
I would be willing to donate tissue samples for research as long as my personal details remained anonymous	117 (45.9)	97 (38.0)	25 (9.8)	13 (5.1)	3 (1.2)
*Use of donated tissue samples*					
If I donated tissue samples:					
I would have no objection to my prostate sample being used for any purpose	100 (40.5)	75 (30.4)	45 (18.2)	21 (8.5)	6 (2.4)
I would want to know my individual result	112 (45.2)	82 (33.1)	38 (15.3)	14 (5.6)	2 (0.8)
I would want to know the overall results	104 (42.1)	89 (36.0)	33 (13.4)	21 (8.5)	0
I would like my prostate sample to be used for the development of new treatments for prostate disease	148 (58.9)	84 (33.5)	13 (5.2)	5 (2.0)	1 (0.4)
I would like my prostate sample to be used for development of new methods of diagnosis of prostate disease	145 (58.2)	82 (32.9)	15 (6.0)	5 (2.0)	2 (0.8)
I would like my samples to be used for nonprofit research	140 (55.8)	83 (33.1)	23 (9.1)	3 (1.2)	2 (0.8)
I would like my samples to be used for profit-making research	57 (22.9)	41 (16.5)	69 (27.7)	37 (14.9)	45 (18.0)
I would not like my sample to be used for extra research studies without my specific consent/permission	77 (31.0)	65 (26.2)	39 (15.7)	52 (21.0)	15 (6.1)
In regard to extra research studies I would trust medical researchers to act in an appropriate and ethical way	117 (46.6)	99 (33.4)	29 (11.6)	2 (0.8)	4 (1.6)
